# Synthesis of Polyhedral Borane Cluster Fused Heterocycles via Transition Metal Catalyzed B-H Activation

**DOI:** 10.3390/molecules25020391

**Published:** 2020-01-17

**Authors:** Ke Cao, Cai-Yan Zhang, Tao-Tao Xu, Ji Wu, Xin-Yu Wen, Wen-Jun Jiang, Mao Chen, Junxiao Yang

**Affiliations:** State Key Laboratory of Environment-Friendly Energy Materials & School of Material Science and Engineering, Southwest University of Science and Technology, 59 Qinglong Road, Mianyang 621010, Sichuan, China

**Keywords:** carborane, heterocycle, B-H activation, polyhedral borane cluster

## Abstract

Aromatic heterocycles are ubiquitous building blocks in bioactive natural products, pharmaceutical and agrochemical industries. Accordingly, the carborane-fused heterocycles would be potential candidates in drug discovery, nanomaterials, metallacarboranes, as well as photoluminescent materials. In recent years, the transition metal catalyzed B-H activation has been proved to be an effective protocol for selective functionalization of B-H bond of *o*-carboranes, which has been further extended for the synthesis of polyhedral borane cluster-fused heterocycles via cascade B-H functionalization/annulation process. This article summarizes the recent progress in construction of polyhedral borane cluster-fused heterocycles via B-H activation.

## 1. Introduction

Icosahedral carboranes are a class of carbon-boron molecular clusters with three dimensional aromaticity analogues to benzene, the features of high boron content, extraordinary thermal and chemical stability, and synthetic flexibility make the carborane derivatives be a kind of important building blocks in functional materials [1,2,3,4], key frameworks in pharmaceuticals [5,6,7,8] and ligands in organometallic chemistry [9,10,11,12,13]. Therefore, the selective functionalization of carboranes has attracted considerable interest from chemists [14,15,16,17,18]. 

In recent years, inspired by the C-H activation for direct functionalization of carbon-based molecules, the transition metal-catalyzed B-H activation has emerged as a powerful strategy for selective functionalization of *o*-carboranes and resulted in much advancement [19,20,21,22,23,24,25,26,27,28,29,30,31,32,33,34,35,36,37,38,39,40]. This succinct synthetic strategy offers an efficient protocol for selective functionalization of carboranes, and lead to a class of previously unavailable three dimensional carborane derivatives.

Aromatic heterocycles are ubiquitous building blocks in bioactive natural products, pharmaceutical and agrochemical industries. Based on the three dimensional aromaticity of carborane analogues to benzene, the carborane fused heterocycles would be potential synthons in designing drug candidates and molecular imaging regents for targeted radionuclide therapy [5,41,42]. This article summarizes the recent progress in construction of carborane-fused heterocycles via transition metal-catalyzed B-H activation, and the synthesis of carborane-fused heterocycles and carbocycles via substitution reaction or cycloaddition reaction with cage C-H bonds not included.

## 2. Synthesis of *o*-Carborane-Fused Heterocycles

Because of the 10 B-H bonds of *o*-carborane are not fully equal and the electrophilic reactivity is reduced in the following order: B(9,12) > B(8,10) > B(4,5,7,11) > B(3,6), which makes the functionalization of specific boron vertex to be a challenging subject [43,44]. Inspired by the transition metal catalyzed C-H activation of arenes and the three dimensional aromaticity of *o*-carborane analogues to benzene, by utilizing the palladium catalyzed electrophilic B-H activation, Cao and coworkers disclosed a selective arylation of B(8)-H and B(9)-H bonds of *o*-carboranes with iodobenzene [45]. Almost at the same time, the palladium-catalyzed intramolecular B-H/C_Ar_-Br coupling for synthesis of *o*-carborane-fused dihydroindenes was reported by Xie group [46]. These pioneering works opened the door for selective functionalization of *o*-carboranes via B-H activation and further extend to polyhedral borane clusters [18].

**Figure 1:**
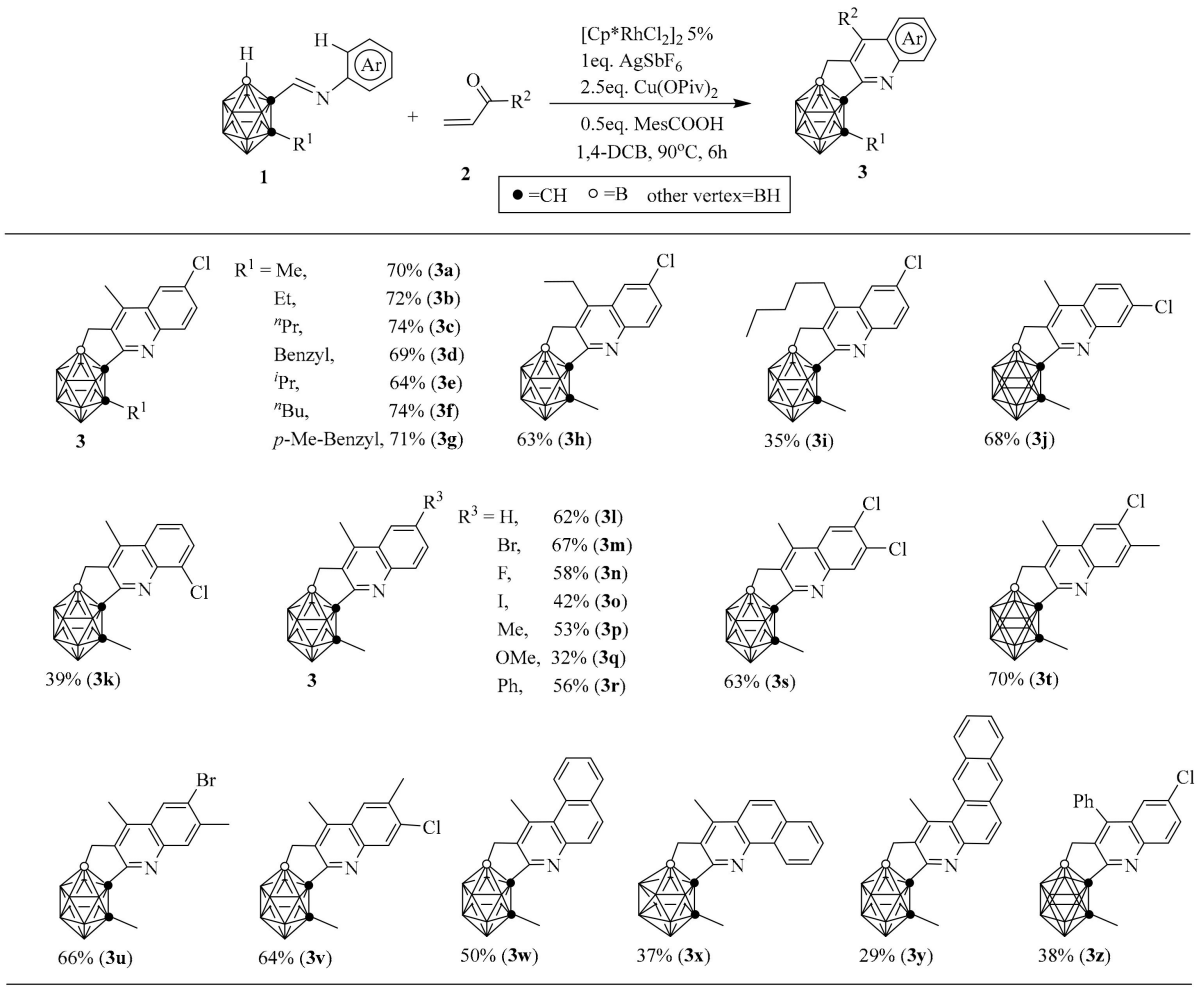
Synthesis of *o*-carborane-fused cyclopenta[*b*]quinolines.

In 2018, Xie group disclosed a rhodium-catalyzed cascade cyclization of carboranyl *N*-arylimines with vinyl ketones for synthesis of *o*-carborane-fused cyclopenta[*b*]quinolines via sequential C-H/B-H activation [47]. This reaction displays well the compatibility for various substituents and gives a series of unprecedented *o*-carborane-fused cyclopenta[*b*]quinolines (Figure 1). 

**Scheme 1:**
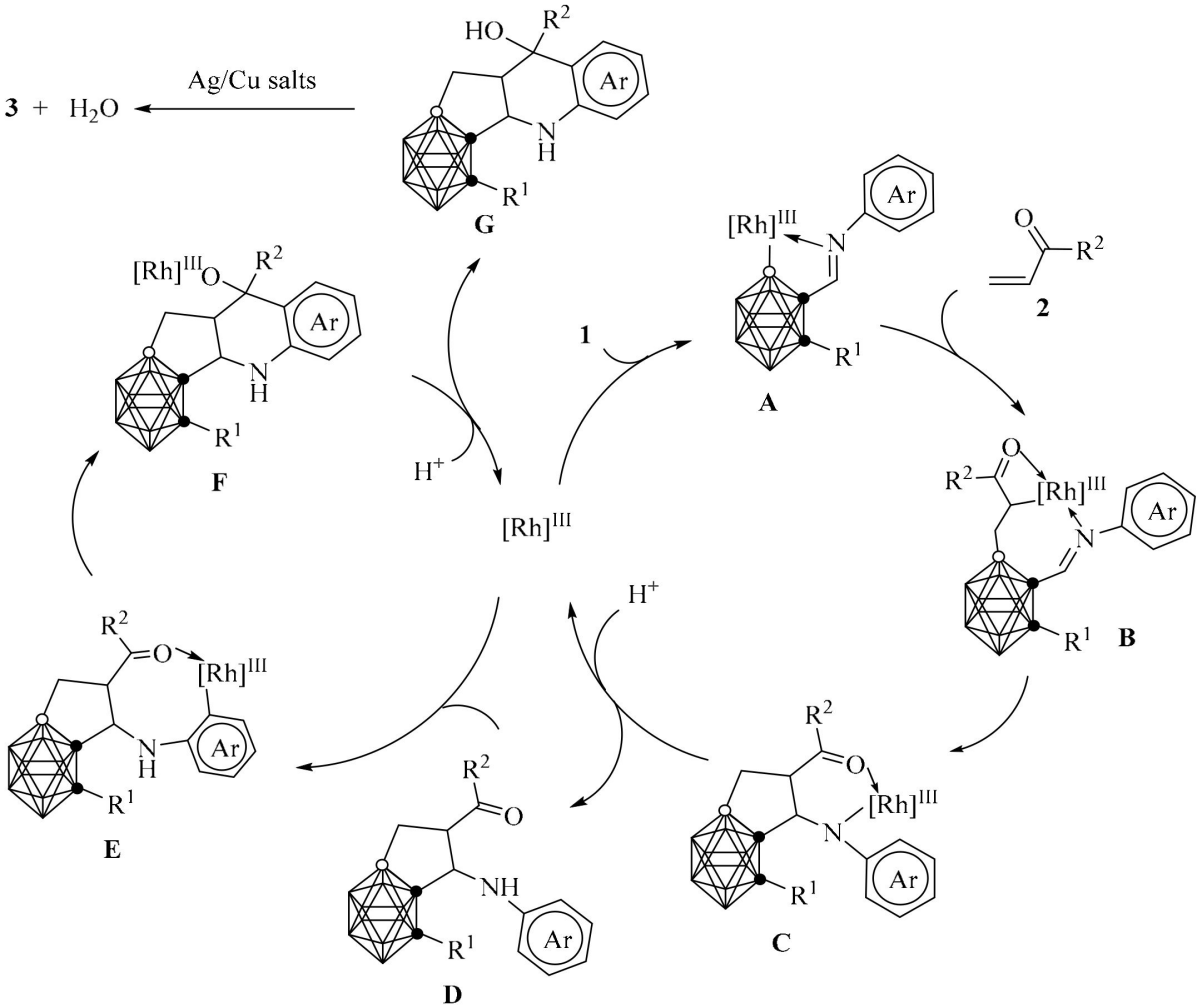
Proposed mechanism for rhodium-catalyzed cascade cyclization of carboranyl *N*-arylimines with vinyl ketones.

Mechanism studies indicate that the activation of cage B-H bond is preferred over the aryl C-H bond by isolating the key intermediate **D**. Based on the control experiment results, a plausible mechanism involving selective B-H activation, alkene insertion, nucleophilic cyclization, C-H activation, nucleophilic cyclization, dehydration and oxidative aromatization was proposed (Scheme 1). 

**Figure 2:**
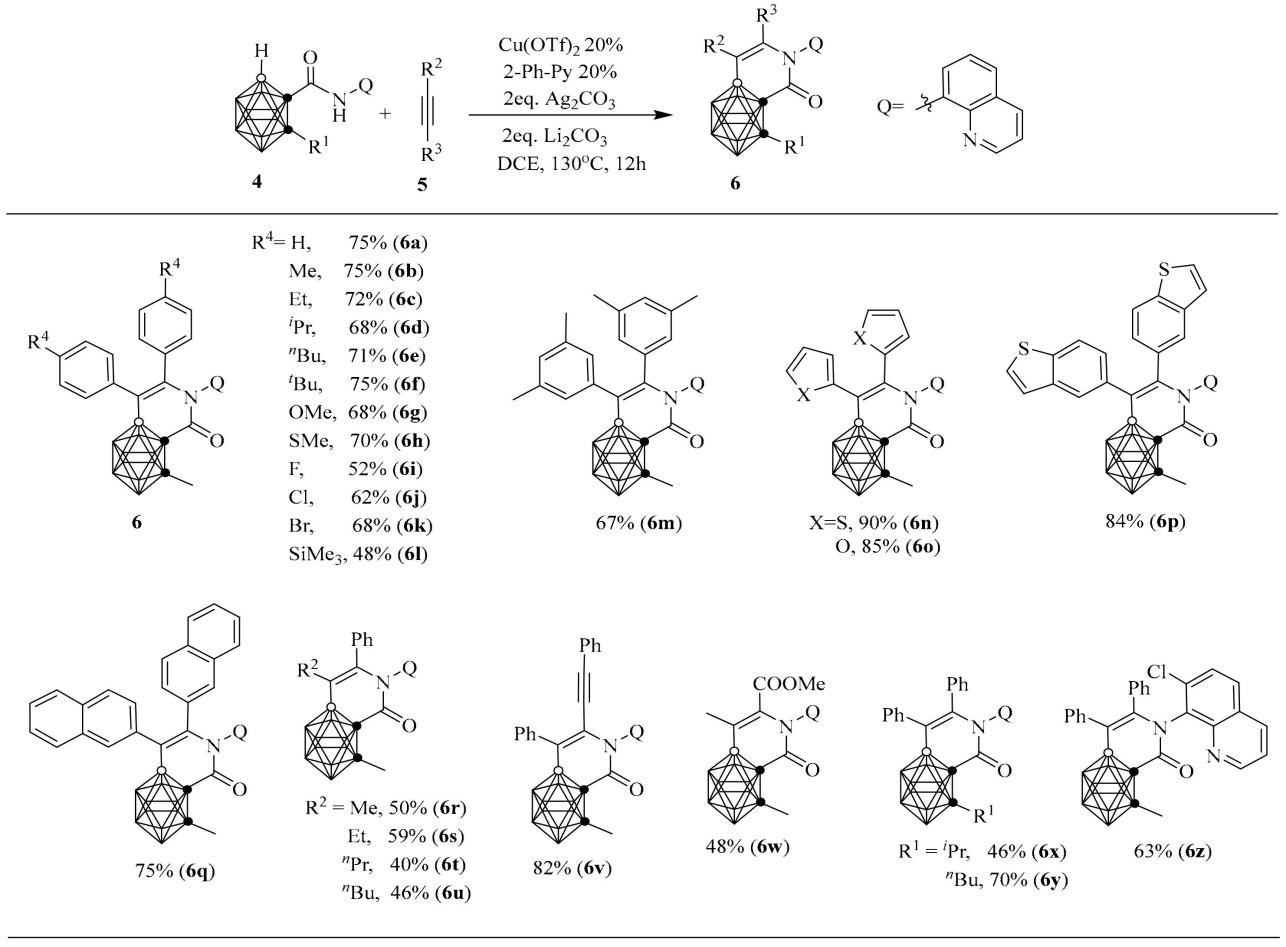
Synthesis of *o*-carborane-fused pyridones.

Based on the directing group guided/transition metal-catalyzed B-H activation for selective functionalization of *o*-carboranes, the copper catalyzed [4+2] annulation of carboranyl amides with internal alkynes for synthesis of *o*-carborane-fused pyridones were accomplished under the assistance of 8-aminoquinoline (Figure 2) [48]. Mechanism studies suggest a Cu^III^-catalyzed B-H activation based on the isolation and structural identification of a stable Cu^I^ intermediate. This is the first example for copper-catalyzed selective B-H activation of *o*-carborane, and offers an alternative catalytic active species for functionalization of *o*-carboranes and boron clusters.

**Figure 3:**
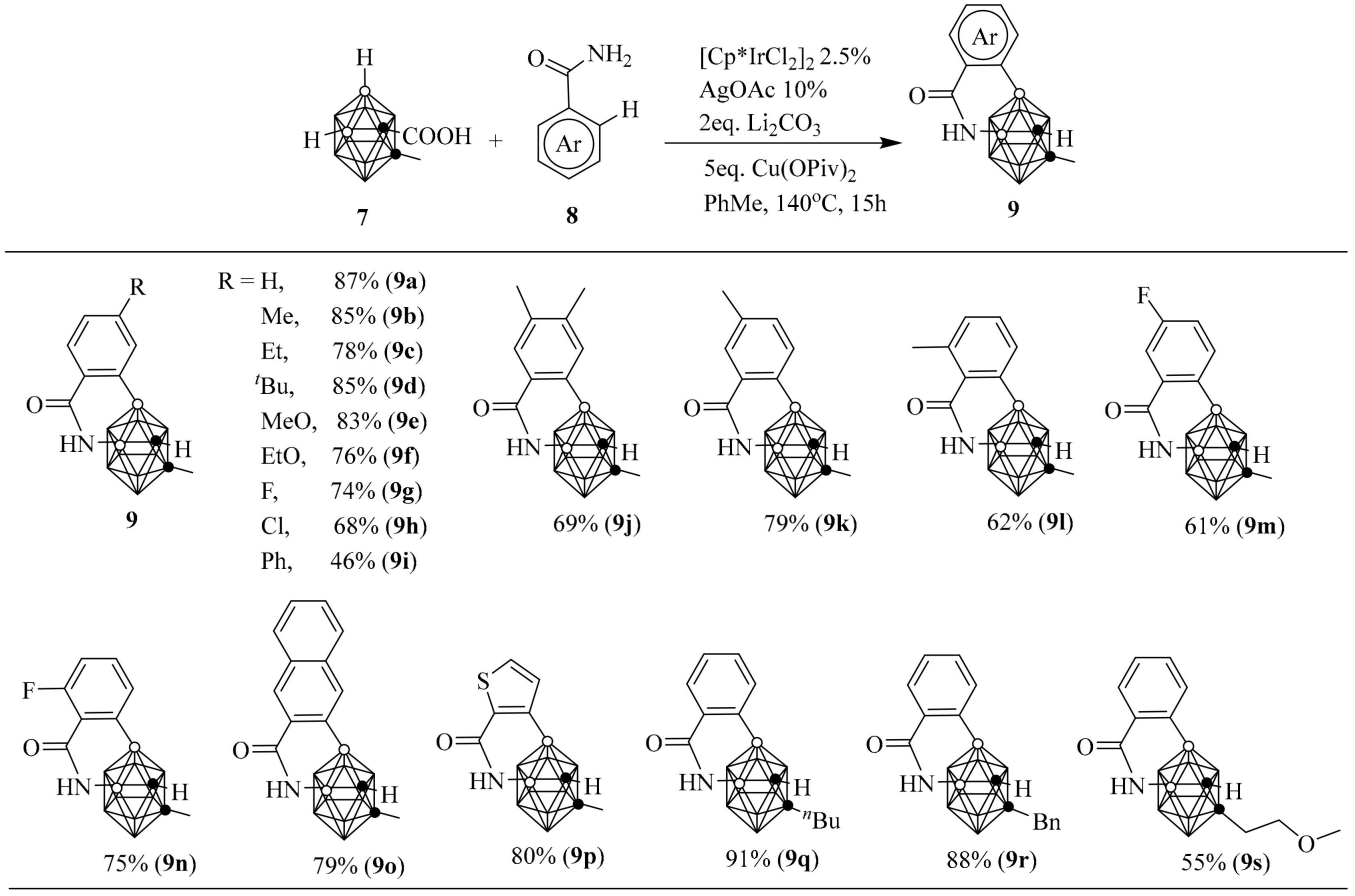
Synthesis of carborano-isoquinolinones.

Recently, by employing a traceless carboxylic acid as directing group, the iridium-catalyzed cascaded dehydrogenative cross coupling of B-H/C-H and B-H/N-H bonds for synthesis of carborano-isoquinolinones was developed in Xie’s group [49]. This reaction represents the most effective approach for synthesis of unprecedented carborano-isoquinolinones with available benzamides and provides important references for construction of *o*-carborane-fused heterocycles via dehydrogenative coupling process (Figure 3). 

This cascade cross coupling could be stopped at the first B-H/C-H coupling step and gives a series of α-carboranyl benzamides, which indicates the forming of B-C bond is preferential over B-N bond. Cyclic voltammetry studies suggest the Ir^V^ should be the key species for the electrophilic C-H metallation and B-N reductive elimination steps, and a mechanism featured with Ir-nitrene species was proposed.

## 3. Synthesis of [CB_11_]^−^-Fused Heterocycle

**Figure 4:**
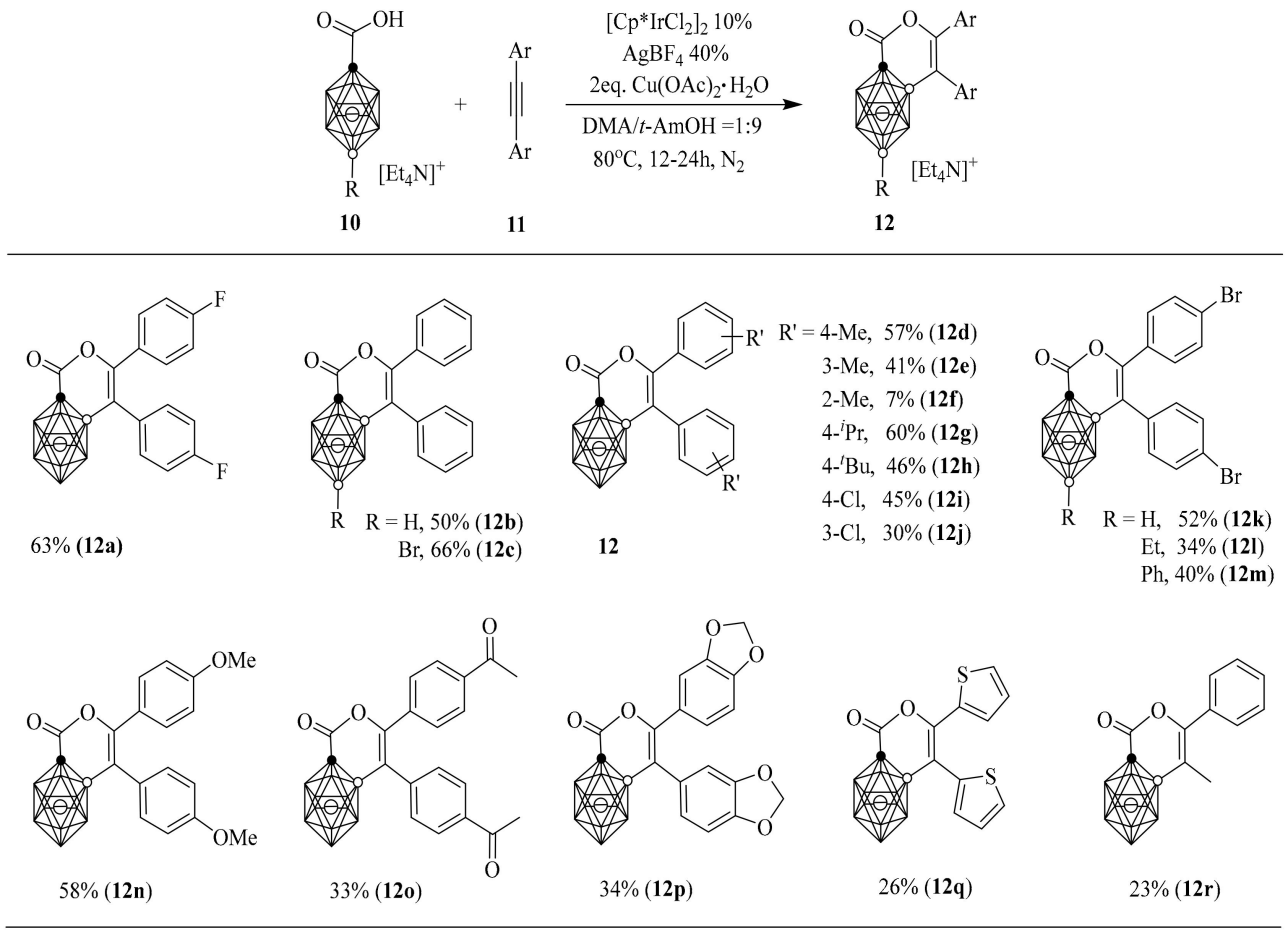
Synthesis of 3D analogues of isocoumarins.

According to the carboxylic acid directed selective B-H activation of *o*-carboranes [30,31,32,33,34,35,36,37], by introducing carboxylic acid into cage carbon of monocarba-*closo*-dodecaborate (CB_11_^−^), the iridium-catalyzed B-H activation/alkenylation/annulation of CB_11_^−^ cluster with diarylacetylenes for synthesis of 3D analogues of isocoumarins was accomplished in Duttwyler’s group [50]. This transformation displays broad substrate scope for kinds of symmetric diarylacetylenes as well as unsymmetric internal alkynes (Figure 4). A carboxylic acid-assisted Ir^I^/Ir^III^ catalytic cycle for B-H activation/alkyne insertion was proposed based on the isolation and control experiments of Ir^III^ intermediate.

## 4. Synthesis of [B_12_]^2−^-Fused Heterocycles

**Figure 5:**
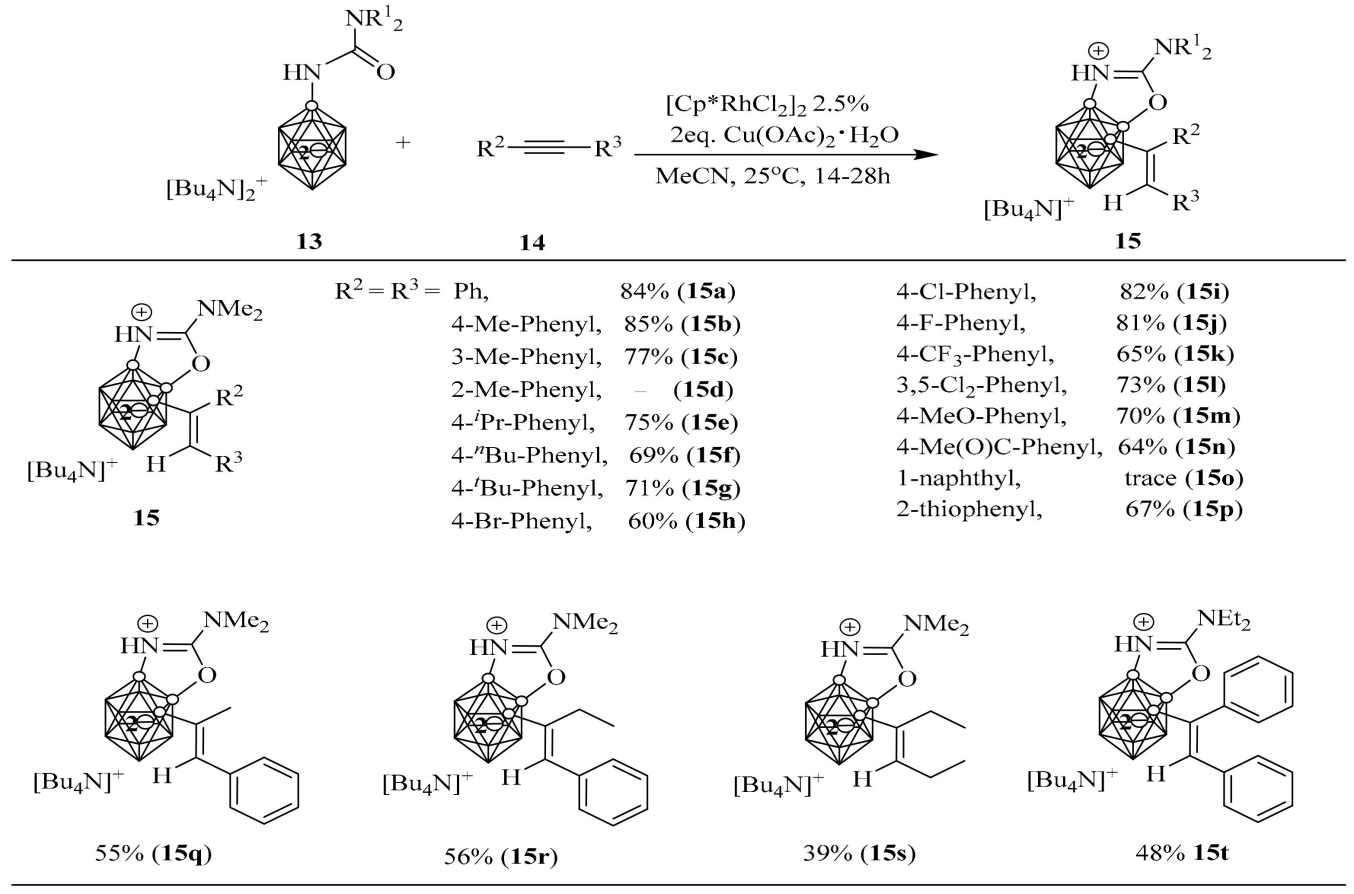
Synthesis of dodecaborate-fused oxazoles.

The dodecahydro-*closo*-dodecaborate dianion [B_12_H_12_]^2−^ is an icosahedral cluster with 12 identical B-H vertices, which make the selective boron functionalization of dodecaborates remains a major synthetic challenge in controlling the degree of substitution and regioselectivity. Traditional methods for B-H functionalization of *closo*-dodecaborates primarily rely on the iodinated precursors [51,52]. According to the transition metal catalyzed B-H activation of *o*-carboranes, Duttwyler and coworkers disclosed a rhodium-catalyzed double B-H activation of dodecaborate anion by employing ureido as directing group for the first time [53], which offers an efficient protocol for the synthesis of dodecaborate-fused oxazoles via one pot alkenylation/annulation process (Figure 5). Mechanism studies indicate the alkenylation occurs prior to annulation in this one pot transformation, and a plausible mechanism involving Rh^I^/Rh^III^ catalyzed dual B-H activation was proposed based on the isolation of a rhodium agostic intermediate.

**Figure 6:**
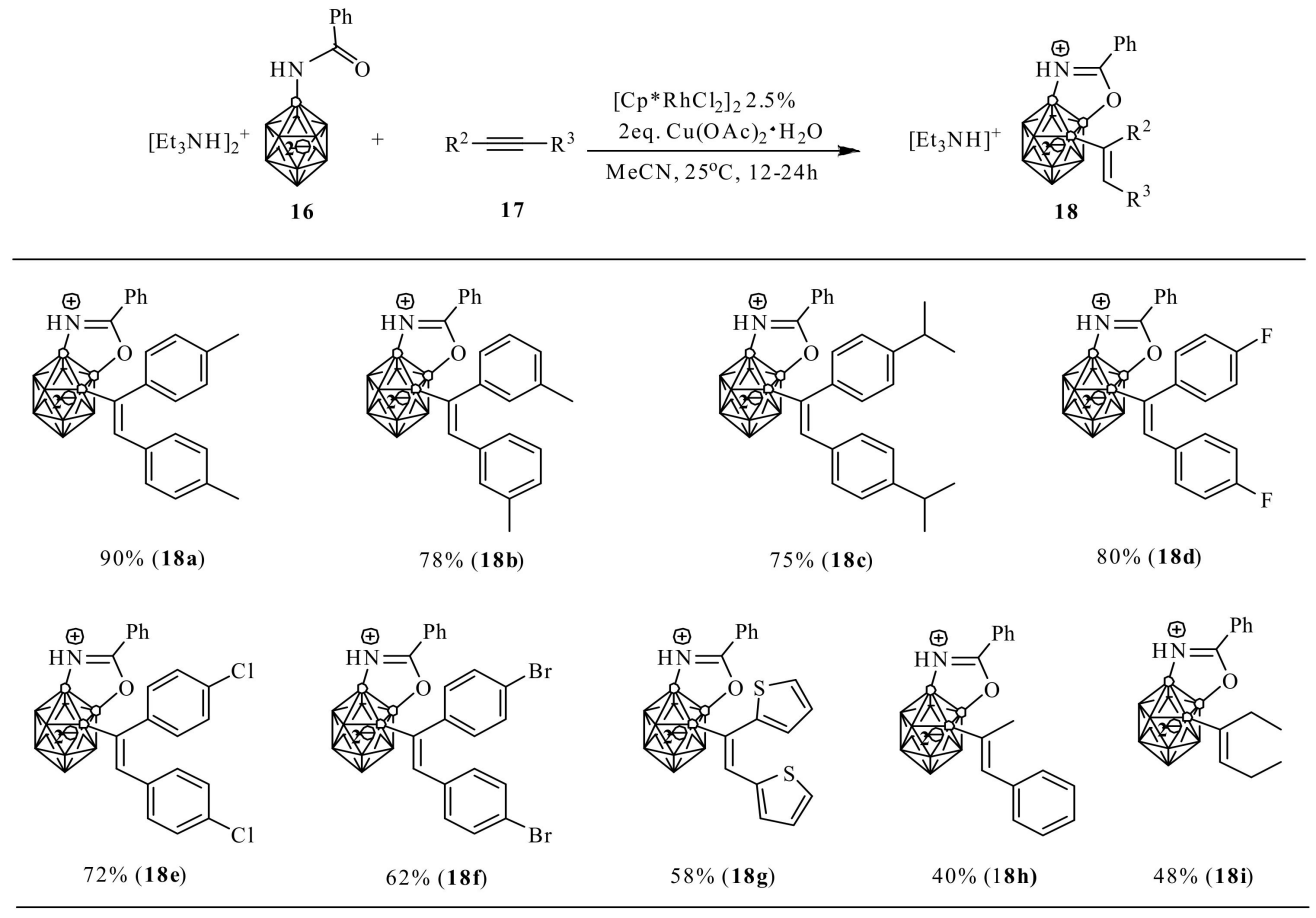
Synthesis of dodecaborate-fused oxazoles with amide as directing group.

Later, the same group further extended the cascade alkenylation/annulation of dodecaborates with amide as directing group [54]. This transformation displays broad functional group tolerance and complete cage regioselectivity, and a series of dodecaborate-fused oxazoles were synthesized with moderate to good yields (Figure 6). Importantly, the above synthesized dodecaborate-fused oxazoles displayed blue emission in solid state, which would be potential candidates for the application in photoluminescent materials. 

**Figure 7:**
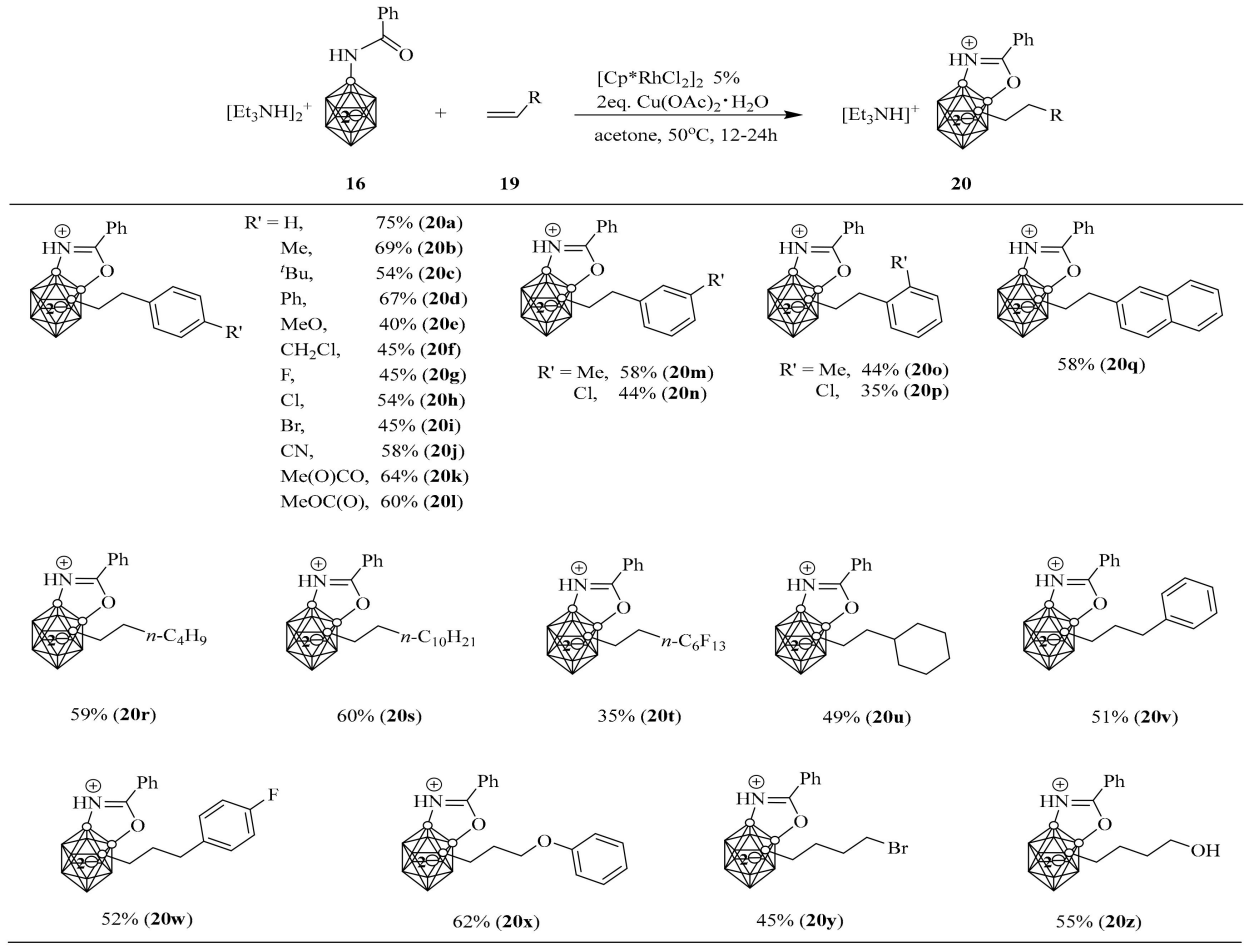
Synthesis of alkyl-substituted dodecaborate-fused oxazoles.

Interestingly, by replacing the alkyne with alkene, the cascade alkylation/annulation of dodecaborates amide was also proceed well in acetone and gives a series of unprecedented alkyl-substituted dodecaborate-fused oxazoles (Figure 7). The resulted products show strong resemblance to antimicriobially active boron clusters, and have potential applications in medicinal chemistry [55].

## 5. Synthesis of *nido*-Carborane-Fused Heterocycles

**Figure 8:**
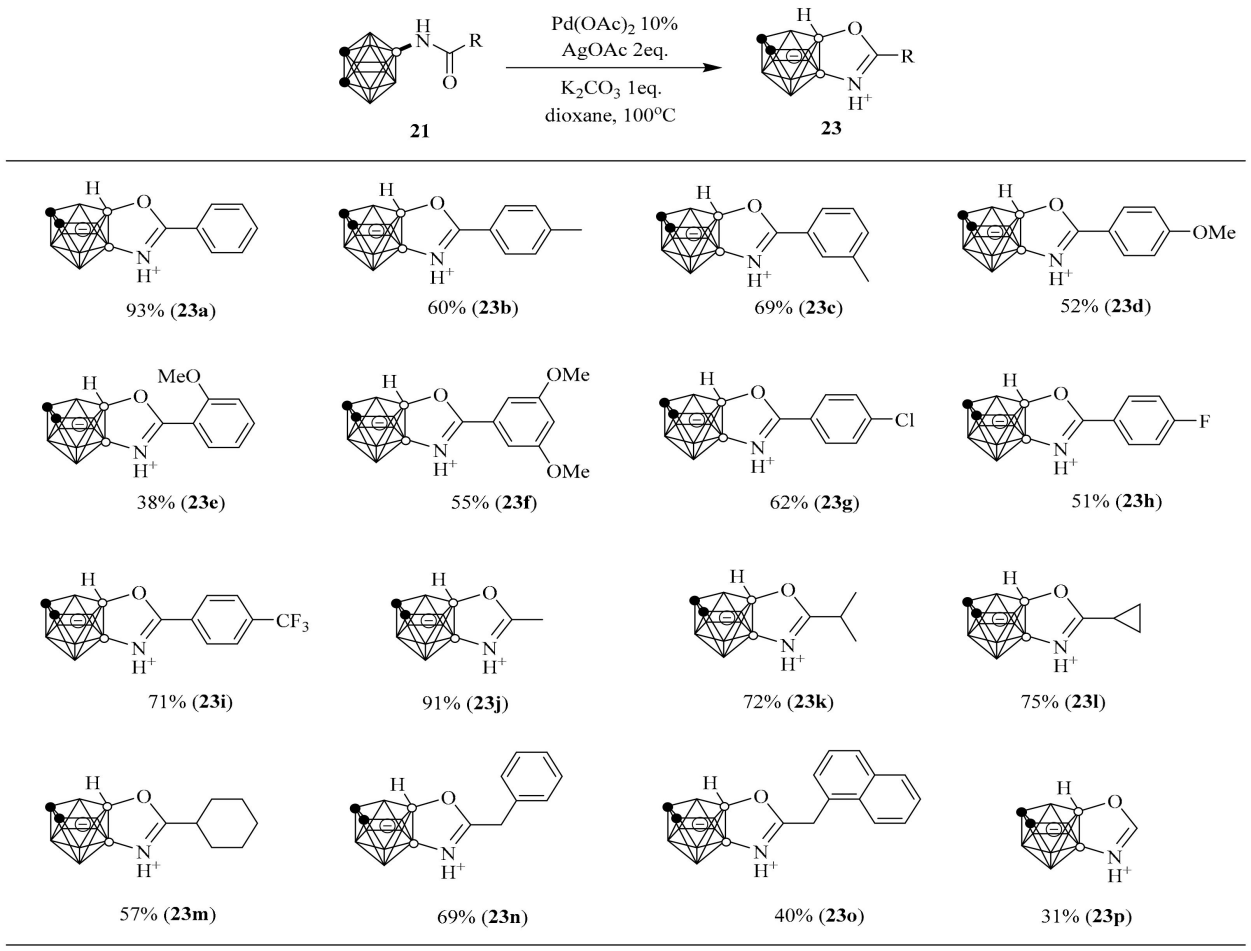
Synthesis of *nido*-7,8-carborane-fused oxazoles.

Recently, by employing amide on cage B(9) of *o*-carborane, an unexpected one pot deboronation/cyclization for the synthesis of *nido*-7,8-carborane-fused oxazole by cooperation of Pd(OAc)_2_, AgOAc, and K_2_CO_3_ has been developed by Cao’s group [56]. A series of unprecedented *nido*-7,8-carborane-fused oxazoles have been synthesized with moderate to good yields (Figure 8), which opens a window for the synthesis of novel kinds of metallacarboranes and targeted radionuclide therapy reagents. 

**Scheme 2:**
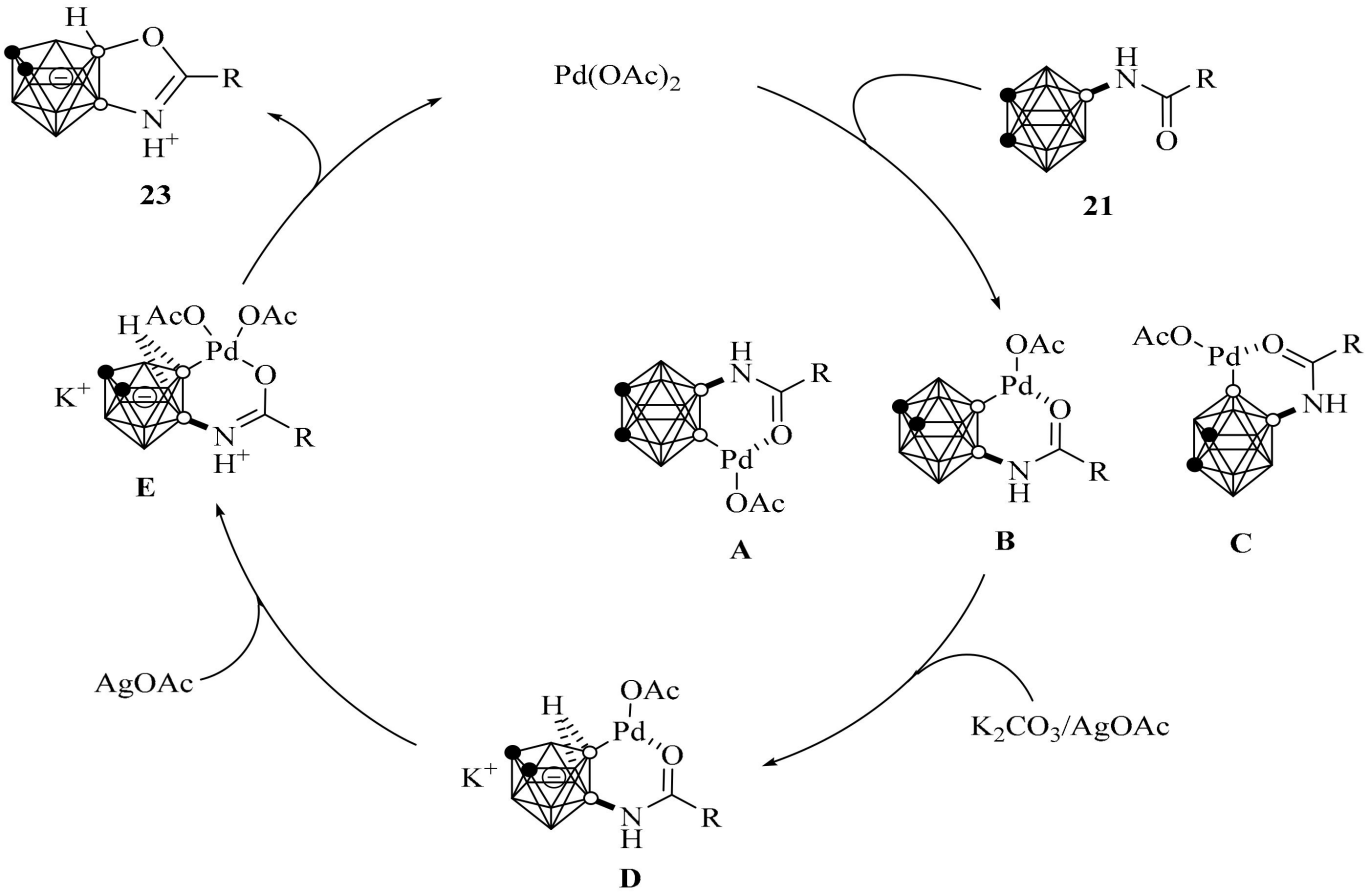
Plausible mechanism for synthesis *nido*-7,8-carborane-fused oxazoles.

To understand the mechanism, they have successfully isolated the key deboronated intermediate and unambiguously characterized by X-ray crystallographic analysis. Control experiments indicate that the deboronation reaction occurs first, and this one pot deboronation/cyclization reaction should be promoted by the cooperative effect of Pd(OAc)_2_, AgOAc, and K_2_CO_3_. Based on these results, a plausible mechanism involving a Pd^II^-catalyzed B(8)-H activation, oxidation, and tautomerization of amide was proposed (Scheme 2). 

## 6. Conclusions

The transition metal-catalyzed B-H activation as a novel synthetic strategy has been proved to be an effective protocol for selective functionalization of B-H bond of polyhedral borane clusters, which has been further extended to synthesis of polyhedral borane cluster-fused heterocycles via cascade B-H functionalization/annulation process. Because of the synthetic flexibility, aqueous stability and general robustness of polyhedral borane clusters, by combining the borane cluster with heterocycles would provide a library of potential candidates in drug discovery, nanomaterials, metallacarboranes, as well as photoluminescent materials. Despite the aforementioned strategies remarkable achievements have been made in recent years, exploring novel cascade reactions for the synthesis of diversified borane cluster-fused heterocycles are still anticipated in the future.
